# Supplementary material for: Three-Dimensional Analysis of Posterior Mandibular Displacement in Rats
Source: Vet Sci. 2022 Mar 20;9(3):144. doi: 10.3390/vetsci9030144 (PMC8953185; doi:10.3390/vetsci9030144)
Supplement: Supplementary file 1 [file vetsci-09-00144-s001.zip › vetsci-1606741-supplementary.pdf]

Supplementary Table S1. Lin's concordance correlation coefficient (rc), Mean difference and Bland–Altman's 95% LOA (Limits of Agreement) between measurements of the first observer.

	rc	p-value	Mean diff.	95% LOA
Go′ - Menton Initial	0.991	<0.001	-0.014	(-0.211, 0.184)
Go - Menton Initial	0.988	<0.001	-0.001	(-0.185, 0.184)
Coronoid - Menton Initial	0.992	<0.001	0.016	(-0.128, 0.160)
Condylion/Go′ - Menton Initial	0.968	<0.001	0.014	(-0.181, 0.209)
Condylion - Go′ Initial	0.979	<0.001	0.010	(-0.148, 0.169)
Condylion - Menton Initial	0.985	<0.001	-0.013	(-0.203, 0.178)
Condylion - Id Initial	0.991	<0.001	-0.010	(-0.167, 0.146)
Condylion - I′ Initial	0.989	<0.001	0.003	(-0.168, 0.174)
Incisal - Id Initial	0.988	<0.001	-0.005	(-0.124, 0.114)
Incisal - I′ Initial	0.992	<0.001	-0.004	(-0.084, 0.076)
Intercondylar Initial	0.856	<0.001	-0.082	(-0.553, 0.389)
Go′ - Menton Final	0.998	<0.001	0.010	(-0.161, 0.181)
Go - Menton Final	0.998	<0.001	0.003	(-0.153, 0.158)
Coronoid - Menton Final	0.998	<0.001	0.032	(-0.105, 0.168)
Condylion/Go′ - Menton Final	0.976	<0.001	0.010	(-0.295, 0.315)
Condylion - Go′ Final	0.978	<0.001	0.017	(-0.284, 0.319)
Condylion - Menton Final	0.998	<0.001	0.000	(-0.155, 0.155)
Condylion - Id Final	0.999	<0.001	-0.001	(-0.131, 0.129)
Condylion - I′ Final	0.998	<0.001	0.004	(-0.163, 0.171)
Incisal - Id Final	1.000	<0.001	-0.005	(-0.124, 0.114)
Incisal - I′ Final	1.000	<0.001	-0.002	(-0.081, 0.076)
Intercondylar Final	0.869	<0.001	-0.147	(-0.480, 0.185)

Supplementary Table S2. Lin's concordance correlation coefficient (rc), Mean difference and Bland–Altman's 95% LOA (Limits of Agreement) between measurements of the two observers.

	rc	p-value	Mean diff.	95% LOA
Go′ - Menton Initial	0.993	<0.001	-0.017	(-0.182, 0.147)
Go - Menton Initial	0.989	<0.001	-0.013	(-0.186, 0.161)
Coronoid - Menton Initial	0.992	<0.001	0.005	(-0.139, 0.148)
Condylion/Go′ - Menton Initial	0.967	<0.001	-0.001	(-0.201, 0.198)
Condylion - Go′ Initial	0.977	<0.001	-0.004	(-0.171, 0.163)
Condylion - Menton Initial	0.986	<0.001	-0.011	(-0.197, 0.174)
Condylion - Id Initial	0.990	<0.001	-0.003	(-0.171, 0.164)
Condylion - I′ Initial	0.989	<0.001	0.010	(-0.163, 0.182)
Incisal - Id Initial	0.980	<0.001	-0.011	(-0.165, 0.143)
Incisal - I′ Initial	0.997	<0.001	-0.003	(-0.052, 0.045)
Intercondylar Initial	0.870	<0.001	-0.050	(-0.486, 0.386)
Go′ - Menton Final	0.999	<0.001	-0.018	(-0.173, 0.137)

Go - Menton Final	0.998	<0.001	-0.008	(-0.178, 0.161)
Coronoid - Menton Final	0.997	<0.001	0.004	(-0.161, 0.169)
Condylion/Go' - Menton Final	0.988	<0.001	0.021	(-0.194, 0.236)
Condylion - Go' Final	0.991	<0.001	0.025	(-0.164, 0.214)
Condylion - Menton Final	0.996	<0.001	-0.006	(-0.213, 0.200)
Condylion - Id Final	0.998	<0.001	0.007	(-0.171, 0.184)
Condylion - I' Final	0.998	<0.001	0.023	(-0.141, 0.186)
Incisal - Id Final	0.999	<0.001	-0.006	(-0.136, 0.123)
Incisal - I' Final	1.000	<0.001	-0.002	(-0.084, 0.080)
Intercondylar Final	0.823	<0.001	-0.133	(-0.603, 0.336)
